# Comprehensive Analysis of Hormonal Signaling Pathways and Gene Expression in Flesh Segment Development of Chinese Bayberry (*Myrica rubra*)

**DOI:** 10.3390/plants14040571

**Published:** 2025-02-13

**Authors:** Yihan Fu, Shuwen Zhang, Li Yang, Yu Zong, Yongqiang Li, Xingjiang Qi, Wenrong Chen, Fanglei Liao, Weidong Guo

**Affiliations:** 1 College of Life Sciences, Zhejiang Normal University, Jinhua 321004, China; fyh0321@zjnu.edu.cn (Y.F.); yangli@zjnu.cn (L.Y.); yzong@zjnu.cn (Y.Z.); lyq@zjnu.cn (Y.L.); cwr@zjnu.cn (W.C.); 2 Institute of Horticulture, Zhejiang Academy of Agricultural Science, Hangzhou 310021, China; hizhangshuwen@163.com (S.Z.); qixj@zaas.ac.cn (X.Q.); 3 Zhejiang Provincial Key Laboratory of Biotechnology on Specialty Economic Plants, Zhejiang Normal University, Jinhua 321004, China

**Keywords:** Chinese bayberry, flesh segments, fruit development, endogenous hormones, transcriptome analysis, paraffin sectioning, immunofluorescence localization

## Abstract

Chinese bayberry (*Myrica rubra* or *Morella rubra*) is a valuable fruit, yet the mechanism of its flesh segment development is not well understood. Using paraffin sectioning, we investigated the flower buds of the ‘Biqi’ and ‘Zaojia’ varieties, revealing that the flesh segment development in these Chinese bayberry varieties involved the formation of a primordium outside the ovary wall, the establishment of a simple columnar structure, and the formation of the primary flesh segment. Assessment of endogenous hormone levels indicated the significant reductions in jasmonic acid (JA) and indole-3-acetic acid (IAA) levels at the critical stages of flesh segment development. Correlation analysis highlighted the essential roles of IAA, JA, abscisic acid (ABA), and gibberellins in the flesh segment developmental process, underscoring the complex interactions driven primarily by the IAA, JA, and ABA networks. Gene modules positively correlated with flesh segment development were identified using transcriptome-based weighted gene co-expression network analysis (WGCNA). Differentially expressed genes (DEGs) were enriched in plant hormone signal transduction pathways, particularly for upregulated genes associated with auxin and JA signaling. Key genes predicted to be involved in flesh segment development included *LAX2* and *LAX3* (auxin transport), *JAZ6* (JA signaling repression), and *KAN1* and *KAN4* (regulating multiple hormonal signaling pathways). Quantitative real-time polymerase chain reaction (qRT-PCR) validation confirmed that the expression trends for these genes were consistent across both varieties, particularly for *CRC*, *SEP1*, *SEP3*, *IAA7*, and *JAZ6*. Immunofluorescence localization studies revealed that auxin was primarily distributed in the central vascular bundle and outer cells of the flesh segment. This uneven auxin distribution might contribute to the unique morphology of flesh segments. Overall, this study provides insights into the hormonal regulation and genetic factors involved in the development of Chinese bayberry flesh segments.

## 1. Introduction

**Figure 1:**
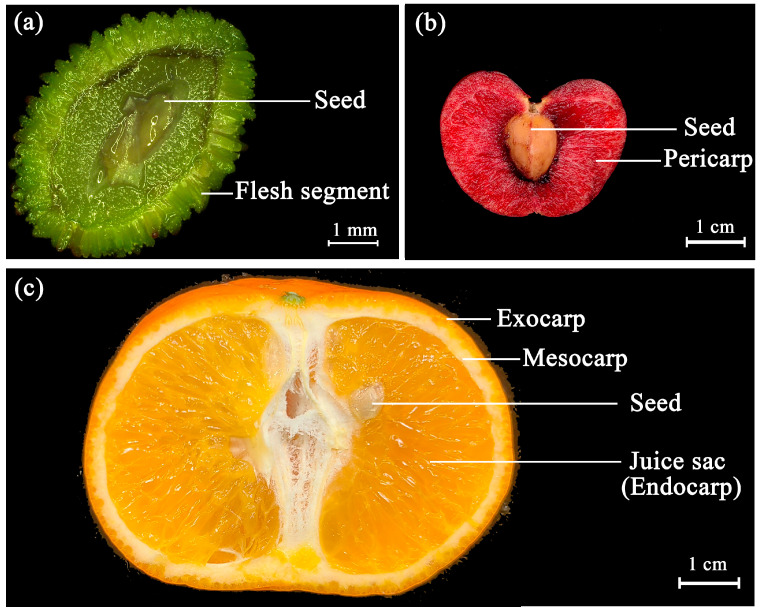
Comparisons of (**a**) bayberry, (**b**) cherry, and (**c**) citrus fruit structures. Bayberry has a unique fruit structure where the outer edge of the pericarp is specialized to form flesh segments. These segments are morphologically similar to the juice sacs of citrus.

Chinese bayberry (*Myrica rubra* or *Morella rubra*; 2n = 16) is a subtropical evergreen fruit tree native to Asia. Renowned for its delicate flesh and unique flavor, it is an economically important fruit crop cultivated in regions south of the Yangtze River in China [1,2]. The bayberry fruit is a spherical drupe, with the outer pericarp tightly arranged around the edge of the fruit, forming saccular protrusions known as “flesh segments” (Figure 1a), which are the main edible part of the fruit [3]. The unique structure of bayberry flesh segments suggests that their developmental process differs from that of typical fleshy fruits such as cherries (*Prunus* spp.) (Figure 1b). However, there are few reports on this unique developmental process.

In most fleshy fruits, pericarp development originates from the ovary wall, with cell division and expansion occurring inward to fill the fruit chamber and form a thick fleshy fruit wall [4]. In the *Citrus* genus, the edible part of the fruit, known as the “juice sac”, consists of multicellular sac-like structures that develop from the inner surface of the ovary (Figure 1c). This development begins within the inner epidermis of the carpel and progresses in three stages. First, the original cells of the juice sac undergo multidirectional division to create a spherical body attached to the inner carpel surface, referred to as the juice sac primordium [5]. Second, continuous cell division in the spherical body elongates it into the carpel cavity, forming a young columnar structure with meristematic properties [6,7]. Third, the cells at the top of the columnar structure continue dividing and elongating, thickening the structure into mature juice sacs [8,9]. Bayberry flesh segments share some similarities with citrus juice sacs: Both are columnar, filled with parenchyma cells, and feature a cap-like structure at the tip [10]. However, unlike juice sacs, which lack vascular tissue, bayberry flesh segments contain vascular bundles that run through them. Moreover, the developmental pattern of juice sacs (filling the fruit chamber from the inner pericarp) differs significantly from that of bayberry flesh segments (extending outward from the outer pericarp). Understanding the unique development of bayberry flesh segments holds significant implications for enhancing existing theories of fruit development.

Fruit development involves intricate morphological and physiological changes, with endogenous hormones playing crucial regulatory roles [11,12,13]. Indole-3-acetic acid (IAA) regulates various aspects of plant growth and development [14]. Frenkel [15] proposed that a reduction in the IAA level is necessary for the maturation of fleshy fruits. Jasmonic acid (JA), a natural plant growth regulator, controls downstream gene expression via the JA signaling pathway during plant growth and development. JA signaling plays a significant role in root elongation, flower development, and fruit ripening [16]. Gibberellic acid also impacts fruit set and enlargement [17,18]. The regulatory mechanism of ethylene in the elongation of fruit shape in *Cucurbitaceae* plants has been elucidated [19,20]. In addition, the important regulatory roles of melatonin and salicylic acid (SA) in fruit ripening and quality have been demonstrated [21,22,23]. Different endogenous hormones have diverse functions and regulatory mechanisms in fruit development, making them worth researching and discussing. Typically, plant hormone regulation involves complex interactions between hormones, forming an intricate regulatory network. This complexity makes it challenging to analyze the function of a single hormone. For example, the application of exogenous and endogenous IAAs in cucumber (*Cucumis sativus* L.) can regulate the levels of endogenous hormones such as gibberellic acid and abscisic acid (ABA), thereby affecting fruit development [24]. Ethylene regulates the fruit ripening process by interacting with plant hormones such as ABA, JA, and IAA [25]. Some researchers have suggested that the coordination among ABA, IAA, and gibberellins in *Arabidopsis thaliana* plays a role in the normal development of the silique [26]. Similarly, the interaction between IAA and ABA in tomatoes (*Solanum lycopersicum* L.) affects fruit shape development [27]. Compared with the relatively well-understood hormonal regulatory network governing flower development, the mechanisms underlying pericarp development in fruits remain poorly studied.

Currently, a systematic description of bayberry flesh segment development is lacking key aspects; the changing levels of endogenous hormones and their interactions during flesh segment development remain unclear. Additionally, studies on the functional genes responding to downstream hormone signaling pathways in bayberry are relatively scarce. Therefore, this study focuses on the ‘Biqi’ and ‘Zaojia’ varieties of *Myrica rubra*. The development of bayberry flesh segments was described using the paraffin sections of bayberry flower buds. The levels of endogenous hormones at different developmental stages of bayberry flesh segments were measured, and transcriptome analysis was conducted to reveal the key physiological changes and molecular mechanisms involved in the development of bayberry flesh segments.

## 2. Results

### 2.1. Morphology of Bayberry Flower Buds and the Development of the Pistil Before Flesh Segment Formation

**Figure 2:**
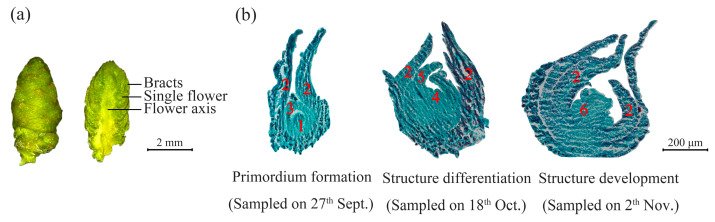
Morphological and histological characteristics of bayberry flower buds. The bayberry variety used for the morphological and histological observations was ‘Biqi’. The flower buds had not reached the peak flowering stage during these observations. (**a**) The longitudinal section of the inflorescence in the bayberry flower buds. (**b**) Paraffin sections showing the development of bayberry flower buds: 1 represents the pistil primordia of the flower bud, 2 represents the flower bud bracts, 3 represents the bract primordia, 4 represents the pistil, 5 represents the stigma, and 6 represents the ovary.

The female bayberry flowers are arranged in racemose inflorescences, with their floral structures enveloped by multiple layers of bracts located between the bracts and the floral axis (Figure 2a). Typically, only 2–4 flowers at the top of the inflorescence develop into fruit [3]. Paraffin sectioning revealed that flower bud development proceeded through three stages before transitioning to flesh segment formation (Figure 2b): the first was the initial formation of female flower primordia, which was characterized by the emergence of pistil and bract primordia; the second was the development of floral structures (including large and small bracts) and the specialization of stylar structures; and the third was the maturation of female flowers, which was characterized by the slight enlargement of the pistil ovary without the development of flesh segments. During this phase, as flesh segment structures had not yet appeared, bayberry flower buds mainly focused on the development of floral structures. Morphologically, the floral axis was relatively short and granular. The bracts of terminal flowers were relatively mature, while the remaining bracts remained tightly attached to the flower bud portion enclosing the pistil.

### 2.2. Development of Flesh Segments in Female Bayberry Flowers: From Primordium Formation to Primary Flesh Segment Establishment

**Figure 3:**
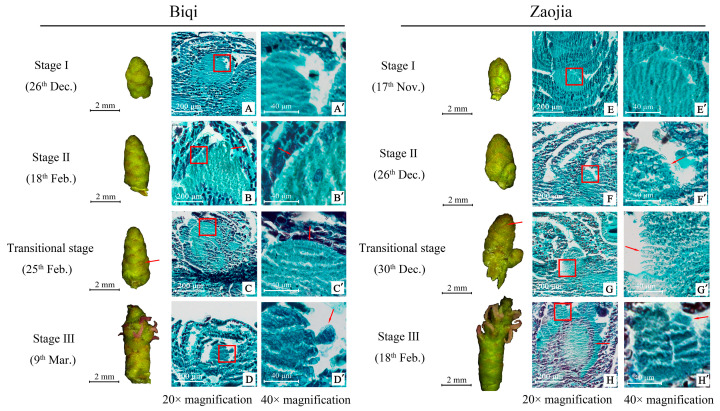
Paraffin sections illustrating the development of bayberry flesh segments. For the ‘Biqi’ variety, the first column shows the appearance of flower buds sampled at different time points (scale bar = 2 mm). (**A**–**D**) display flower bud sections under a 20× magnification (scale bar = 200 μm), while (**A’**–**D’**) provide observations of the red-boxed areas in (**A**–**D**) under a 40× magnification (scale bar = 40 μm), with the arrows indicating the protruding structure of the flesh segment primordia or primary flesh segment. For the ‘Zaojia’ variety, the first column shows the appearance of flower buds sampled at different time points (scale bar = 2 mm). (**E**–**H**) show flower bud sections under a 20× magnification (scale bar = 200 μm), while (**E’**–**H’**) provide observations of the red-boxed areas in (**E**–**H**) under a 40× magnification (scale bar = 40 μm), with the arrows indicating the protruding structure of the flesh segment primordia or primary flesh segment. In the first stage of flesh segment development, the ovary structure of the pistil expanded.

The biological process of flesh segment development in the ‘Biqi’ and ‘Zaojia’ bayberry varieties was documented (Figure 3). The flesh segment originated from the outer layer of cells in the swollen ovary wall, forming flesh segment primordia. In the ‘Zaojia’ variety, the ovary began to swell around 17 November (Figure 3E), with the flesh segment beginning to develop by 26 December (Figure 3F), approximately 40 and 55 days earlier than the ovary swelling initiation and flesh segment morphology establishment in the ‘Biqi’ variety, respectively. Both varieties exhibited densely packed cells on the outer wall of the swollen floral bud ovary (indicated by the red rectangles in Figure 3B,F). These larger cells, featuring thorn-like structures protruding outward to fill the gap between the ovary and bracts (Figure 3B’,F’), contained multiple nuclei (which were deeply stained), suggesting active cellular processes and mitotic potential. They were identified as the primordial cells of the flesh segment.

Bayberry inflorescence observations during this period revealed that stylar structures remained intact (indicated by the arrow labeled “stylar structure” in Figure 3B), indicating that flesh segment formation occurred during flower-to-fruit transformation. This suggested that normal floral organ development was crucial for flesh segment formation, which accompanied the ongoing development of floral structures. The cells in the flesh segment primordia further divided and formed a regularly arranged layer of cells on the outer ovary wall (indicated by the red rectangles in Figure 3C,G). Under magnification, these cells appeared larger and plumper than primordial cells, and they had no visible vacuoles (Figure 3C’,G’), indicating the formation of the primary flesh segment. This stage represented a transitional phase in flesh segment development. During this stage, the flesh segments began to demonstrate a columnar shape, indicating the initial manifestation of their morphology.

As floral buds reached the flesh segment initiation stage, they elongated, and the bracts matured. The edges darkened, implying increased lignification (indicated by the arrows in the super-depth-of-field images of ‘Biqi’ on 25 February (Figure 3C’) and ‘Zaojia’ on 30 December (Figure 3G’)). This elongation resulted in the further enlargement of female bayberry floral buds due to the formation of the primary flesh segment.

The flesh segment primordia continued to grow and elongate, forming rod-like structures. During this stage, the cell walls of small flesh segment cells thinned, and their cytoplasm lightened, indicating that they were predominantly parenchyma cells. In Figure 3D, a typical young flesh segment is indicated by an arrow. Notably, the top of the small flesh segment is wider than the base, with thinner cell walls and more transparent cells, suggesting a lack of nutrients and pigments. These immature cells exhibited strong meristematic potential, facilitating further elongation.

By 9 March, further development in the ‘Zaojia’ variety (Figure 3H’) revealed a mostly established flesh segment shape. A ring of deeper-stained cells inside the ovary (indicated by the arrows in Figure 3H) was likely to develop into the seed coat, marking the initial formation of the bayberry fruit.

In both variants, their flesh segment development shows the same pattern. In the second stage, the primordial cells of the flesh segment were produced, marking the initiation of flesh segment development. In the transitional stage, these primordial cells developed into columnar flesh segment primordia. In the next stage, the flesh segment primordia developed into a primary flesh segment. Based on paraffin section observations, the development of bayberry flesh segments was divided into three stages (Figure 3). In Stage I (26 December for ‘Biqi’ and 17 November for ‘Zaojia’), no flesh segment development was observed. In Stage II (18 February for ‘Biqi’ and December 26 for ‘Zaojia’), the formation of flesh segment primordial cells was noted. In Stage III (9 March for ‘Biqi’ and 18 February for ‘Zaojia’), the formation of the primary flesh segment was documented.

### 2.3. Fluctuations in Endogenous Hormone Levels During Flesh Segment Development

**Figure 4:**
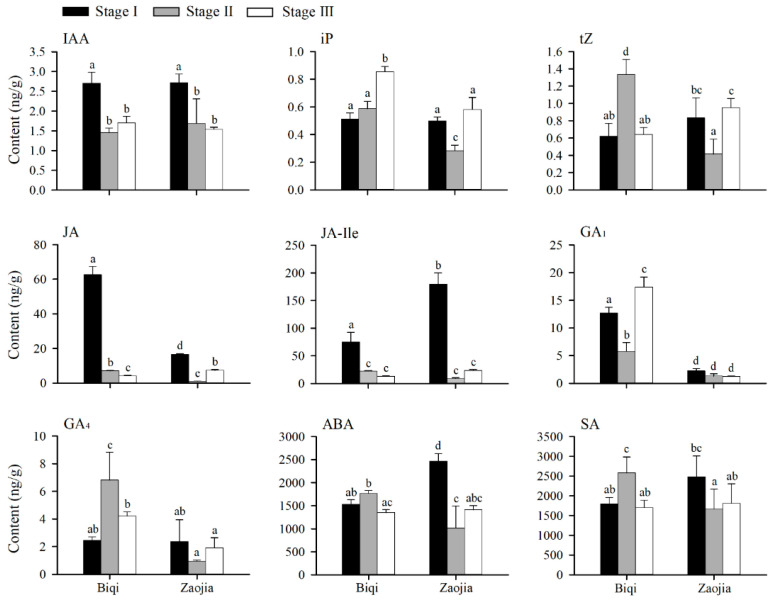
Fluctuations of the endogenous hormone levels in the flower buds of ‘Biqi’ and ‘Zaojia’ during flesh segment development. Samples for Stage I were collected before the initiation of flesh segment development, on 26 December for ‘Biqi’ and 17 November for ‘Zaojia’. Samples for Stage II were collected at the stage of flesh segment primordial cell formation, on 18 February for ‘Biqi’ and 26 December for ‘Zaojia’. Samples for Stage III were collected after the formation of the primary flesh segment, on 9 March for ‘Biqi’ and 18 February for ‘Zaojia’. Significant differences were analyzed using Duncan’s test, with different lowercase letters indicating significant differences (*p* < 0.05). In both ‘Biqi’ and ‘Zaojia’, the levels of JA, JA-Ile, and IAA were significantly downregulated at the initiation of flesh segment development. In ‘Zaojia’, the level of JA decreased substantially (by approximately 93.83%) after primordium formation. Meanwhile, in ‘Biqi’, the level of iP increased significantly during the subsequent stages of flesh segment development. In contrast, ‘Zaojia’ exhibited an initial decrease in the iP level, followed by a recovery. However, the iP level in ‘Zaojia’ remained lower than that in ‘Biqi’.

The levels of endogenous hormones in the flower buds of the ‘Biqi’ and ‘Zaojia’ bayberry varieties were analyzed across the three stages of flesh segment development. Notably, ‘Biqi’ exhibited significant fluctuations in hormone levels, particularly during the transition from Stage I to Stage II (Figure 4). In contrast to the level of iP (isopentenyladenine, cytokinins), the levels of auxin, gibberellin GA_1_, JA, and jasmonic acid-isoleucine (JA-Ile; the active form of JA) decreased markedly, with JA displaying the most substantial reduction (approximately 88.70%). In the ‘Biqi’ variety, the level of tZ (trans-Zeatin, cytokinins) was significantly upregulated at the initiation of flesh segment development, but it subsequently decreased to the level observed before flesh segment development. The level of SA increased significantly after the formation of flesh segment primordial cells, suggesting its role in enhancing stress resistance during this crucial phase.

From Stage II to Stage III, only the levels of iP and gibberellin GA_1_ increased, indicating their roles in cell elongation and proliferation within the flesh segment. Interestingly, while the level of JA decreased significantly, that of JA-Ile remained stable, implying that elevated JA levels impeded flesh segment development.

In the ‘Zaojia’ variety, plant hormone levels declined significantly after flesh segment primordium formation, with the JA level decreasing by up to 93.83%, consistent with the trend observed in ‘Biqi’. Both varieties showed similar patterns of reduced JA-Ile, IAA, and JA levels during the early stage of flesh segment development, which stabilized at lower levels in the later stage. However, upon primary flesh segment formation, ‘Zaojia’ exhibited a slight increase in the JA level, which potentially facilitated its early flowering and enhanced its stress resistance.

The level of iP in ‘Zaojia’ decreased at the initial stage of flesh segment primordium formation but recovered in the later stage, with the iP levels in ‘Zaojia’ remaining consistently lower than those in ‘Biqi’. Moreover, in contrast to ‘Biqi’, ‘Zaojia’ exhibited decreasing levels of gibberellins GA_1_ and GA_4_. However, the levels of SA and ABA in ‘Zaojia’ remained stable across different stages of flesh segment development. These observations suggested the distinct regulatory mechanisms of endogenous hormones in the flesh segment development of these two bayberry varieties.

Overall, the significant fluctuations in hormone levels surrounding the emergence of the flesh segment primordia indicated the importance of endogenous hormones and their signaling pathways in bayberry development.

### 2.4. Hormonal Crosstalk During Flesh Segment Development

**Figure 5:**
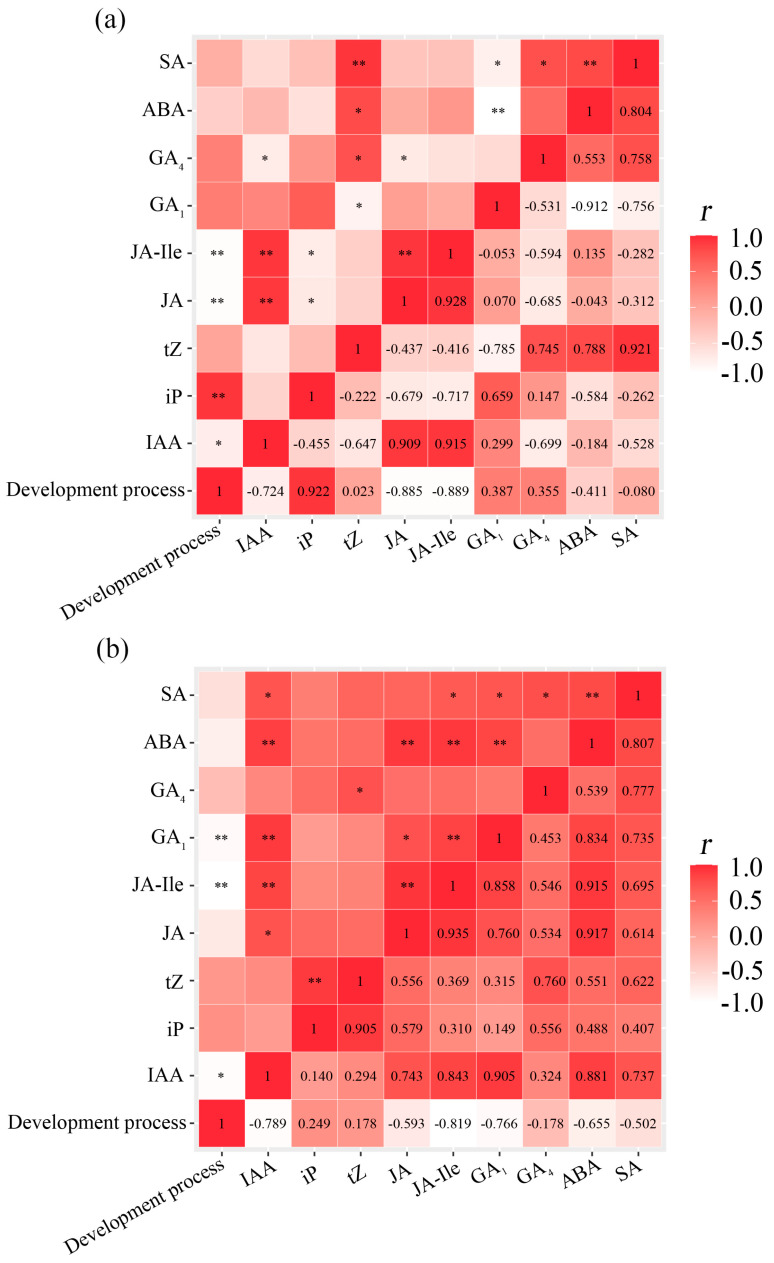
Correlations between flesh segment development stages and endogenous hormone levels. Pearson’s correlation analysis was conducted, and the numerical values in the figure represented correlation coefficients (*r* values). Positive or negative values indicate the direction of the correlations, while asterisks denote the significance of the correlations (* *p* < 0.05 and ** *p* < 0.01). In both (**a**) ‘Biqi’ and (**b**) ‘Zaojia’, the degree of flesh segment development was negatively correlated with the levels of IAA, JA, and JA-Ile. Moreover, there were significant correlations among the levels of these three hormones throughout the flesh segment development process.

Pearson’s correlation analysis was used to analyze the correlations between the changes in endogenous hormone levels and the stages of flesh segment development. This analysis also provided a preliminary understanding of the potential hormonal regulatory networks involved in the flesh segment development process. Different values were assigned to specific stages in the development of bayberry flesh segments: 1 for the stage before the initiation of flesh segment development, 2 for the stage at which the formation of flesh segment primordial cells was initiated, and 3 for the stage at which the primary flesh segment was formed. Pearson correlation analysis (Figure 5a) revealed significant negative correlations between the developmental stage of ‘Biqi’ flesh segments and the levels of IAA, JA, and JA-Ile, with particularly strong correlations for JA (*r* = −0.724 *), JA-Ile (*r* = −0.885 **), and IAA (*r* = −0.889 **). Conversely, positive correlations were observed between the developmental stage of flesh segments and the levels of gibberellins (GA_1_ and GA_4_) and cytokinins (iP and tZ), with iP showing the strongest correlation (*r* = 0.922 **). These results indicated the crucial roles of IAA, JA, JA-Ile, and iP in bayberry flesh segment development.

For the ‘Zaojia’ variety (Figure 5b), strong correlations were identified between the flesh segment development stage and the levels of IAA (*r* = −0.789 *), JA-Ile (*r* = −0.819 **), and GA_1_ (*r* = −0.766 *). The ‘Zaojia’ variety exhibited pronounced relationships among hormones, with significant positive correlations of IAA with JA-Ile (*r* = 0.843 **) and GA_1_ (*r* = 0.905 **), along with positive correlations between ABA and other hormones, including JA and gibberellins.

In both varieties, the flesh segment development stage was inversely related to the levels of IAA, JA, and JA-Ile, indicating that lower hormone levels were associated with more advanced developmental stages. Notably, the differences in hormonal interactions observed between the two varieties suggested the existence of distinct regulatory networks influencing flesh segment development. Overall, IAA, JA, and ABA were identified as the key regulators in the development of bayberry flesh segments.

### 2.5. Transcriptome Acquisition and Preliminary Statistical Analysis of Differentially Expressed Genes

After sequencing quality assessment (Table S1), 120.33 Gb of clean data was obtained, with each sample having at least 5.72 Gb of clean data. The Q30 base percentage was 96.05% or higher. The samples were aligned to the bayberry reference genome, showing an alignment efficiency range of 92.92–97.55%, confirming that the data were suitable for bioinformatics analysis.

The transcriptome data were validated by performing qRT-PCR analysis on selected genes using the internal standard method (2^−ΔΔCt^). The obtained relative expression levels were compared to transcriptome FPKM values (Figure S1). A total of 12 genes related to plant hormone signaling pathways were selected, and their relative expression levels were measured and calculated for the ‘Biqi’ and ‘Zaojia’ varieties. The expression trends of most of these genes were consistent with the transcriptome data, showing an 83.3% similarity. This consistency demonstrated the reliability of the transcriptome data, confirming their suitability for subsequent analysis.

**Figure 6:**
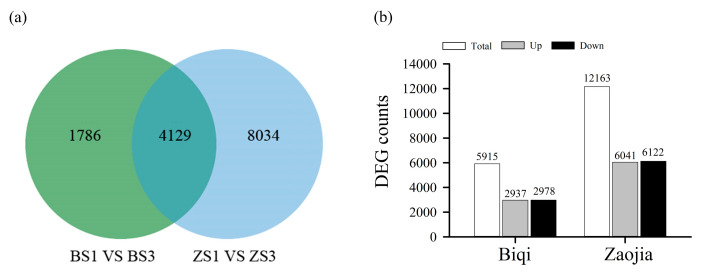
Statistics on the number of DEGs before and after flesh segment development. (**a**) A Venn diagram on the number of DEGs. (**b**) A bar chart on the number of upregulated and downregulated DEGs.

DESeq2 was used to identify differentially expressed genes (DEGs) between ‘Biqi’ and ‘Zaojia’ samples at different stages of flesh segment development (*FDR* < 0.01, |Fold Change| > 1.0). Through a comparison of gene expression levels between Stage I and Stage III, 5915 DEGs were identified in the ‘Biqi’ variety (BS1 vs. BS3), and 12,163 DEGs were identified in the ‘Zaojia’ variety (ZS1 vs. ZS3). Among these, 4129 DEGs were shared between the two varieties during flesh segment development (Figure 6a). In the ‘Biqi’ variety, 2937 genes were upregulated and 2978 genes were downregulated. In comparison, in the ‘Zaojia’ variety, 6041 genes were upregulated and 6122 genes were downregulated (Figure 6b).

### 2.6. Analysis of Gene Co-Expression Networks and Identification of Key Members in Modules Related to Flesh Segment Development

**Figure 7:**
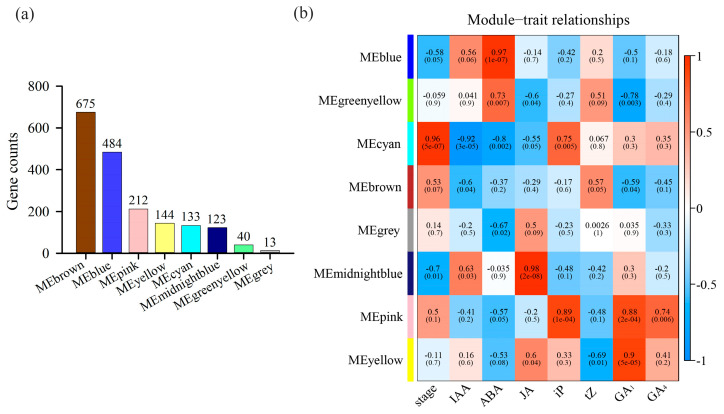
WGCNA analysis of genes identified in the transcriptome data on bayberry flesh segment development. (**a**) Statistics on the number of genes in each module. (**b**) A heatmap showing the associations between WGCNA co-expression modules and traits related to bayberry flesh segment development. The MEcyan, MEblue, and MEmidnightblue modules were significantly correlated with the development of bayberry flesh segments.

After the transcriptome data were analyzed, missing, outlier, and abnormal values were excluded, resulting in the retention of 1826 genes with expression values greater than 1. The optimal network connectivity was achieved at a soft threshold of 18, and gene co-expression network analysis was performed using weighted gene co-expression network analysis (WGCNA). Genes with similar expression patterns were grouped into eight distinct co-expression modules, each identified by a unique color. Based on the gene expression levels, a correlation-based clustering analysis was performed. The MEBrown module contained the largest number of genes, with 675 genes, while the MEGrey module contained the fewest genes, with only 13 genes (Figure 7a). Correlation analysis was performed among the eight modules, bayberry flesh segment developmental traits, and plant hormone levels (Figure 7b). The MEcyan module exhibited a highly significant positive correlation with flesh segment development, while the MEblue and MEmidnightblue modules showed significant negative correlations with flesh segment development. Further analysis revealed that the gene co-expression levels in the MEcyan module were significantly or highly significantly correlated with the levels of auxin, ABA, JA, and iP. Specifically, the gene co-expression levels in the module were negatively correlated with the levels of auxin, ABA, and JA while showing a positive correlation with the level of iP. The gene co-expression levels in the MEblue module showed a significant positive correlation with the ABA level, while the gene co-expression levels in the MEmidnightblue module were significantly or highly significantly positively correlated with the levels of auxin and JA.

In the MEcyan module, which was positively correlated with flesh segment development, annotation using the Pfam and Swiss-Prot databases identified genes involved in plant hormone signaling pathways and floral organ development. A protein interaction prediction network for the 133 members of the module was constructed using STRING (https://cn.string-db.org/), and K-means clustering was performed, resulting in nine color-coded clusters (Figure S2). There were two clusters containing a significant number of proteins, and their roles in floral meristem determination, cytokinin signal transduction, and cytokinin metabolism pathways were inferred by functional annotation. Analysis of the most densely connected clusters identified key proteins in the regulation of carpel development and fruit shape formation, including HEC2, JAG, SEP1, SEP3, and CRC. The first four proteins are known to influence carpel shape, while the last one plays a regulatory role in tissue radial elongation and carpel polarity differentiation [28,29,30,31]. These proteins closely interacted with those involved in plant hormone signaling pathways, including methyltransferase JMT in the JA signaling pathway [32], CYP94B1 in the JA-Ile oxidation degradation pathway [33], early response proteins AUX/IAA14 and SAUR5 in the auxin signaling pathway [34], auxin transport protein LAX3 [35], regulatory protein GASA1 [36], and gibberellin oxidase GA20OX1 in the gibberellin signaling pathway [37]. These findings suggested that auxin and JA signal transduction regulated the expression of genes associated with carpel development, such as *CRABS CLAW* (*CRC*) and *SEPALLATA* (*SEP*), during flesh segment development.

### 2.7. Gene Set Enrichment Analysis (GSEA) of DEGs Involved in Flesh Segment Development

When |NES| > 1, *p*-value < 0.05, and *q*-value < 0.25, the 20 most significantly enriched gene sets in the ‘Biqi’ and ‘Zaojia’ varieties were selected according to their *p*-values. The results are presented in Table 1 and Table 2. In the ‘Biqi’ variety, pathways such as anatomical structure morphogenesis (GO:0009653), plant organ morphogenesis (GO:1905392), post-embryonic plant morphogenesis (GO:0090698), and organic acid transport (GO:0015849) showed a coordinated gene expression upregulation trend during flesh segment development. In the ‘Zaojia’ variety, pathways such as carpel development (GO:0048440), plant-type ovary development (GO:0035670), post-embryonic plant organ morphogenesis (GO:0090697), and specification of plant organ identity (GO:0090701) showed relatively high enrichment. Additionally, pathways related to the temperature response, including response to temperature stimulus (GO:0009266) and response to heat (GO:0009408), were highly enriched. This indicated that the ‘Zaojia’ variety was more sensitive to environmental temperature changes, potentially contributing to its early flowering and fruiting traits. Within the background gene set, the members of the gene sets were represented by black lines. The enrichment results for the ‘Biqi’ variety showed a higher number of core genes enriched in pathways related to anatomical structure morphogenesis and plant organ morphogenesis (Figure S3a). The ‘Zaojia’ variety exhibited a greater number of genes enriched in the post-embryonic plant organ morphogenesis pathway (Figure S3b).

**Figure 8:**
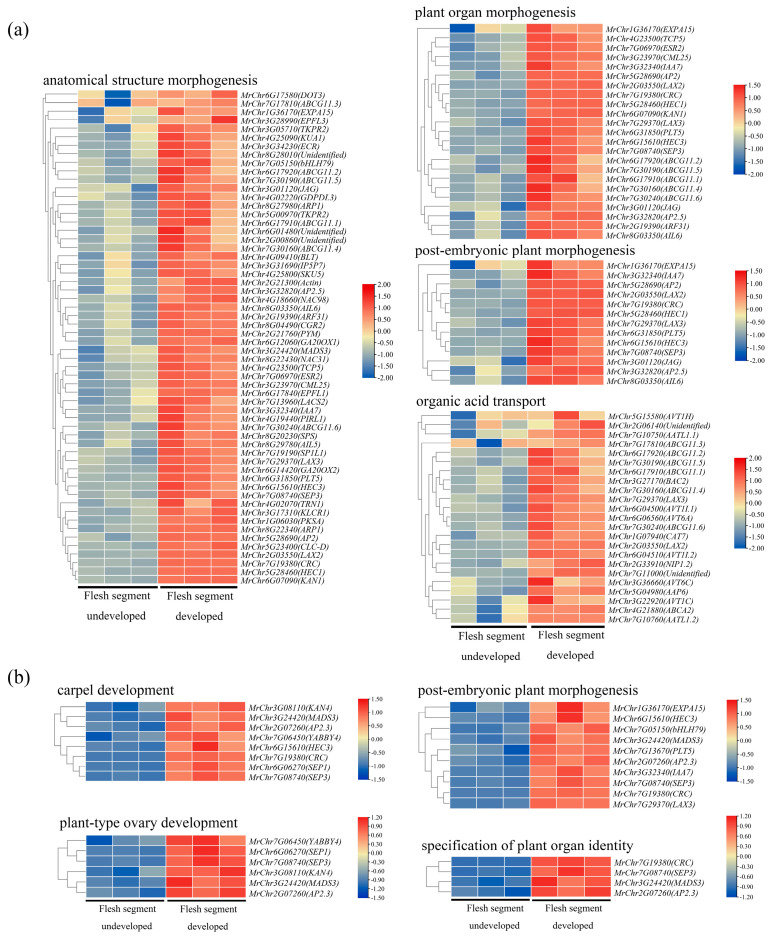
A heatmap of the core gene expression levels in the biological pathways enriched in (**a**) ‘Biqi’ and (**b**) ‘Zaojia’. The identified core genes included genes involved in plant hormone signaling pathways, such as *LAX3* and *IAA7*, as well as genes involved in pistil development, including *CRC*.

The core members of the gene sets identified above were used to plot a heatmap of gene expression levels before and after flesh segment development in both varieties (Figure 8). The identification of core members in the two related pathways of the two varieties revealed that genes regulating pistil development, such as *CRC* (MrChr7G19380), *SEP1* (MrChr6G06270), *SEP3* (MrChr7G08740), show significant upregulation in expression after the development of the flesh segment. Notably, there is active expression of genes related to auxin and JA pathways. For example, members of the *ABCG* family involved in jasmonic acid transport and members of the *LAX* family involved in auxin transport show significant upregulation. Additionally, some response factors in hormone signal transduction, such as the early auxin response gene *IAA7* (MrChr3G32340) and *AP2* family members involved in downstream responses of the jasmonic acid pathway, also exhibit similar upregulation trends. Based on the expression trends of the core members in the pathways related to ‘Biqi’ (Figure 8a) and ‘Zaojia’ (Figure 8b), a similarity in the molecular regulatory mechanisms underlying flesh segment development was inferred between the two varieties. Genes involved in carpel development were actively expressed and might play a shaping role in carpel morphology. JA and auxin, along with genes related to their transport, were actively expressed during flesh segment development. At the same time, downstream genes in these two signaling pathways also showed a cooperative upregulation trend. It was speculated that the plant regulated the levels and distribution of JA and auxin to control plant hormone signaling pathways and biological processes such as flesh segment morphogenesis.

### 2.8. Kyoto Encyclopedia of Genes and Genomes (KEGG) Pathway Enrichment and Expression Pattern Analysis of DEGs Involved in Flesh Segment Development

**Figure 9:**
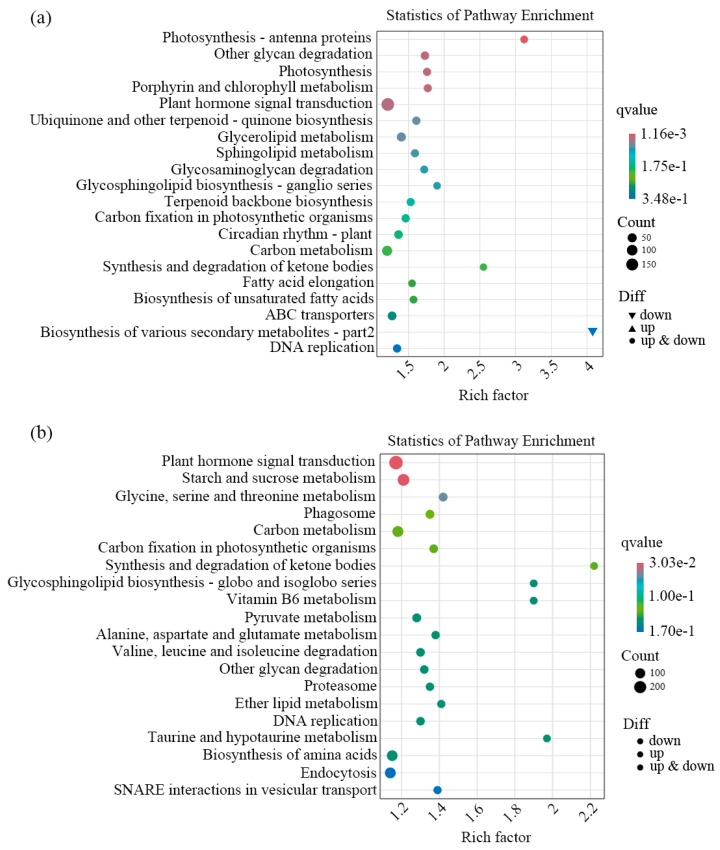
KEGG pathway enrichment analysis of DEGs involved during flesh segment development in (**a**) ‘Biqi’ and (**b**) ‘Zaojia’. These plant hormone signaling pathways were highly enriched during the flesh segment development process.

KEGG pathway enrichment analysis was performed on the DEGs involved during flesh segment development in the ‘Biqi’ and ‘Zaojia’ varieties, presenting the top 20 pathways with the smallest *q*-values for significance (Figure 9). In both varieties, the plant hormone signal transduction pathway was the most significantly enriched, containing the largest number of DEGs. In the ‘Biqi’ variety, DEGs were enriched in the photosynthesis and glycan degradation pathways. Meanwhile, in the ‘Zaojia’ variety, they were enriched in the starch and sucrose metabolism pathways.

DEGs upregulated in the plant hormone signal transduction pathways involved during flesh segment development in the ‘Biqi’ and ‘Zaojia’ varieties were selected through KEGG classification, and a heatmap of gene expression levels was generated, resulting in the identification of 51 and 70 upregulated DEGs in ‘Biqi’ and ‘Zaojia’, respectively.

**Figure 10:**
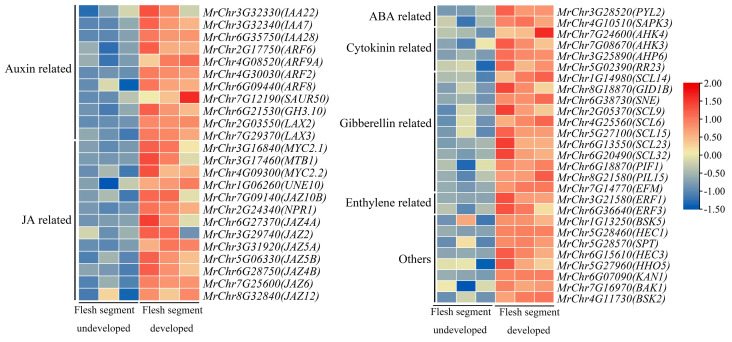
Heatmap analysis of the DEGs upregulated in the plant hormone signaling pathways involved during flesh segment development in ‘Biqi’.

Genes related to the JA and auxin signaling pathways were the predominant genes in ‘Biqi’ (Figure 10). There were 13 upregulated DEGs in the JA signaling pathway, mainly including JA pathway inhibitor genes from the *JAZ* family (e.g., MrChr6G27370, MrChr7G25600, and MrChr5G06330) and downstream JA response genes from the *MYC2* family (e.g., MrChr3G16840 and MrChr3G17460). The upregulated DEGs in the auxin signaling pathway mainly included early auxin-responsive genes from the *AUX/IAA* family (e.g., MrChr6G35750 and MrChr3G32340), members of the *GH3* family (e.g., MrChr6G21530), auxin response factors from the *ARF* family (e.g., MrChr6G09440 and MrChr4G08520), and the auxin influx transporter genes *LAX2* and *LAX3*. In the gibberellin signaling pathway, members of the *GRAS* (*DELLA*) family showed significantly higher expression levels after flesh segment development. Additionally, genes involved in hormone-regulated carpel development were identified, including MrChr5G28460, MrChr5G28570, and MrChr6G07090. Notably, *KAN1* (MrChr6G07090), which regulates the polarity and morphogenesis of distal tissues in plants, was also detected.

**Figure 11:**
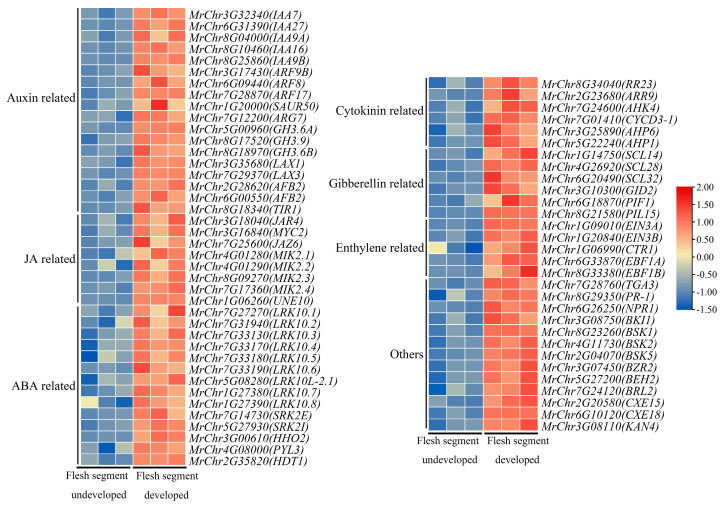
Heatmap analysis of DEGs that were upregulated in the plant hormone signaling pathways involved during flesh segment development in ‘Zaojia’.

Compared to the ‘Biqi’ variety, the ‘Zaojia’ variety (Figure 11) exhibited fewer genes related to the JA signaling pathway. However, it is noteworthy that *JAZ6* (MrChr7G25600), a member of the *JAZ* family, showed upregulated expression in ‘Zaojia’. Additionally, *JAR1*, a gene involved in JA-Ile synthesis, was identified in the JA signaling pathway. In both varieties, many members of the *AUX/IAA* and *GH3* families displayed upregulated expression in the auxin signaling pathway. Notably, *IAA7*, *ARF8* (MrChr3G17430), and *LAX3* were upregulated in both varieties. In the ‘Zaojia’ variety, many members of the *LRK10* family (e.g., MrChr7G27270 and MrChr5G08280) showed upregulated expression in the ABA signaling pathway. Researchers have suggested that these genes promote flowering and early fruit ripening [38]. Further investigation is needed to determine whether these genes contribute to the early flowering and fruiting traits in ‘Zaojia’. Additionally, the expression of *KAN4* (MrChr3G08110), a member of the *KAN* family, was upregulated in ‘Zaojia’.

Comparisons of the expression levels of genes related to plant hormone signaling pathways before and after flesh segment development indicated that *JAZ6* from the JA signaling pathway, *IAA7* from the auxin signaling pathway, *LAX3* involved in auxin transport, and *KAN* family members *KAN1* and *KAN4* (which respond to signals from plant hormones) played important roles in the morphological development of bayberry flesh segments.

### 2.9. Examination of the Expression Levels of Genes Potentially Involved in Flesh Segment Development

**Figure 12:**
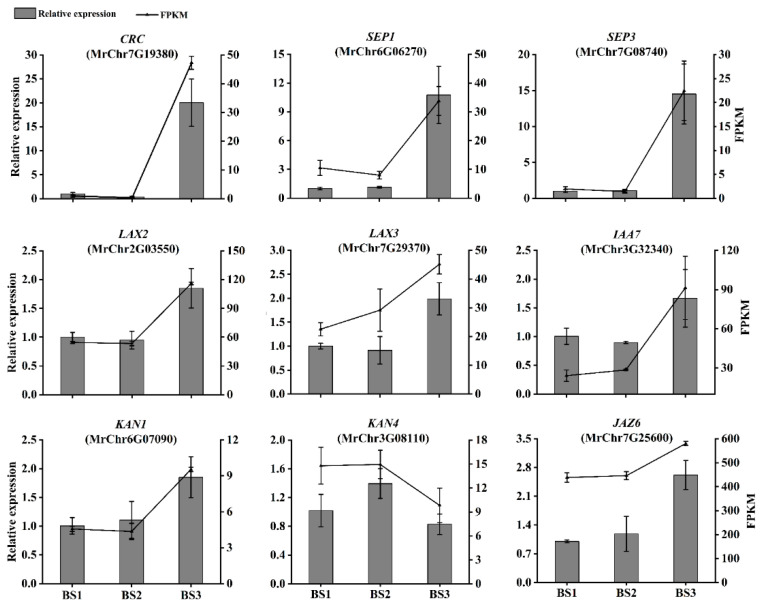
Relative expression levels of genes related to flesh segment development in ‘Biqi’.

**Figure 13:**
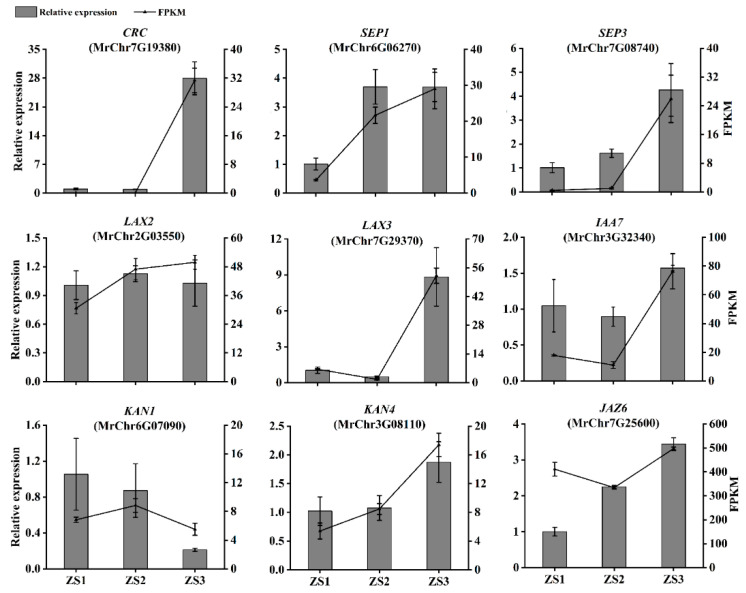
Relative expression levels of genes related to flesh segment development in ‘Zaojia’.

The relative expression levels of genes potentially involved in flesh segment development were analyzed by qRT-PCR and quantified using the internal reference (2^−ΔΔCt^) method. Across the three stages of bayberry flesh segment development, the changes in the expression levels of most genes were consistent with the FPKM changes in transcriptome data. In the ‘Biqi’ variety (Figure 12), the changes in the expression levels of *CRC*, *SEP1*, *SEP3*, *LAX2*, *IAA7*, *KAN1*, and *JAZ6* were largely consistent with the FPKM changes in transcriptome data. Similarly, in the ‘Zaojia’ variety (Figure 13), the changes in the expression levels of *CRC*, *SEP3*, *IAA7*, *LAX3*, and *KAN4* were mostly consistent with the FPKM changes in transcriptome data.

### 2.10. Spatial Distribution of Auxin in Flesh Segments

**Figure 14:**
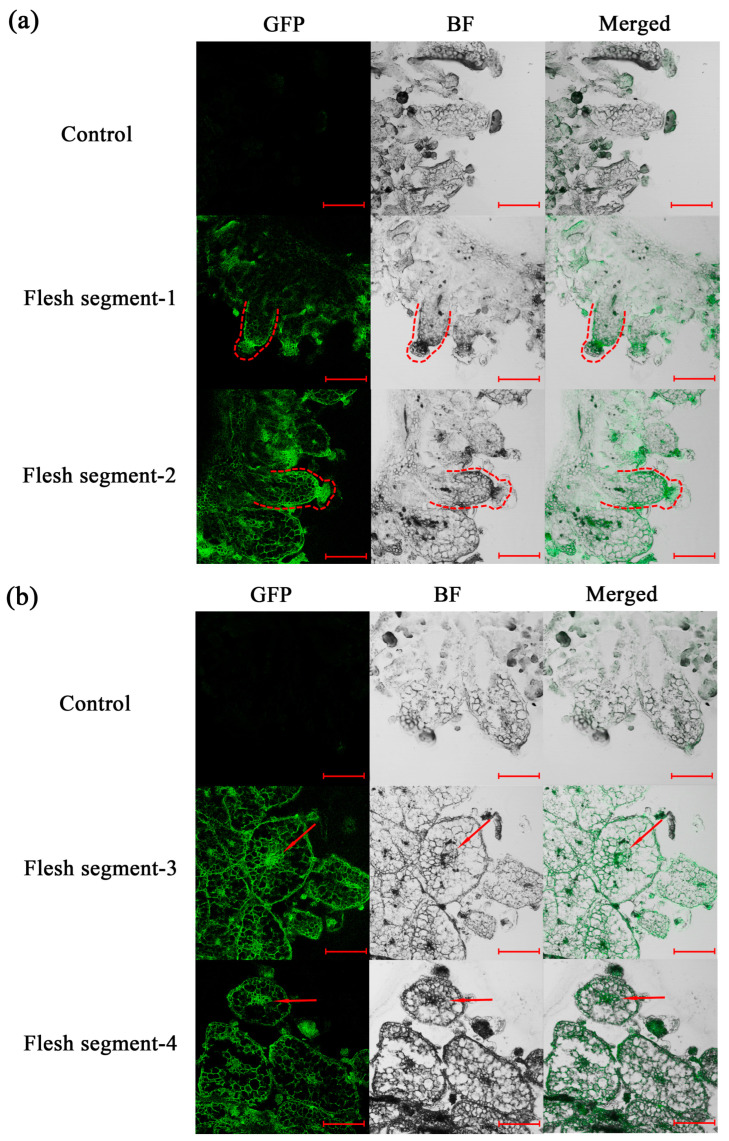
Immunofluorescence localization of auxin in the (**a**) longitudinal and (**b**) transverse sections of flesh segments. The bayberry variety used for the immunofluorescence localization analysis was ‘Biqi’, and all scale bars were set to 200 μm. In (**a**), the dashed line indicates a longitudinal section of a single flesh segment, where the auxin fluorescence signals are enriched at the top and side walls of the flesh segment, forming a continuous linear distribution along the contour marked by the dashed line. In (**b**), the arrow indicates the central vascular bundle within the cross-section of the flesh segment, where the enrichment of auxin fluorescence signals is observed.

Transcriptome data revealed that the expression of genes associated with auxin transport was significantly upregulated during flesh segment development. Therefore, an auxin-specific antibody was used to perform immunofluorescence imaging on the frozen sections of bayberry flesh segments. The spatial distribution of auxin was characterized by the generated fluorescence signal. A negative control, incubated only with a secondary antibody, showed no significant fluorescence signal. Follow-up observations were conducted under these laser excitation conditions. After dual-antibody incubation, laser confocal microscopy was used to observe the longitudinal and transverse sections of the flesh segment. The fluorescence signal of auxin was concentrated at the apex and outer layer of the flesh segment. In the longitudinal section, a strong linear fluorescence distribution was observed, as indicated by the dashed line annotation (Figure 14a). Meanwhile, in the transverse section (Figure 14b), auxin accumulation in the vascular tissue at the center of the flesh segment was detected, as indicated by the red arrow. The uneven distribution of auxin might play a key role in shaping the unique structure of the flesh segment. It is speculated that after synthesis, auxin is transported by the central vascular tissue to the apex of the flesh segment and then evenly distributed across the cell layers of its side walls. The high concentration of auxin on the side walls of the flesh segment hinders the growth of the side wall cells, thereby limiting the lateral expansion of the flesh segment. As basal cells continued to grow, the flesh segment elongated and gradually formed a slender columnar structure.

## 3. Discussion

### 3.1. Morphogenesis and Hormonal Regulation in Bayberry Flesh Segment Development

This study investigated the morphology of bayberry flower buds and the physiological processes involved during flesh segment development in the ‘Biqi’ and ‘Zaojia’ varieties. The flesh segment development involved distinct morphological changes and variations in endogenous hormone levels, highlighting the crucial role of hormonal regulation in flower-to-fruit transformation. Fleshy fruits typically develop from flowers or inflorescences, but variations exist. For instance, tomatoes and grapes (*Vitis vinifera* L.) develop from ovaries, whereas apples (*Malus pumila Mill*.) and strawberries (*Fragaria × ananassa* Duch.) are formed through the enlargement of receptacles or sepals [13].

Studies on the bayberry flesh segment have primarily focused on its origin. This segment comprises the outer pericarp and parts of the middle pericarp, with its developmental source believed to be the ovary wall. However, the exact mechanism of its differentiation remains unclear [10]. Studies have provided further insights into the mechanism of bayberry flesh segment development, revealing similarities with juice sac development in citrus. While the cytological development process remains relatively conserved, involving the formation of primordial cells followed by cell division and differentiation to achieve the final morphology, distinct differences exist between the two. In citrus, juice sacs differentiate inward to fill the locule but are eventually constrained by spatial limitations, hindering further cell proliferation [8]. Conversely, the bayberry flesh segment originates from the outer cell layer of the ovary wall, allowing for fewer spatial limitations on pericarp development. This distinction raises the question of whether bayberry possesses greater potential to cultivate large-fruited varieties compared to citrus.

The differentiation of bayberry flesh segment cells occurs in three stages: primordium formation, simple columnar body development, and primary flesh segment production. Observations of bayberry flower buds at the same developmental stage reveal that the emergence and morphological development of the flesh segment precede the peak flowering period by approximately 30–50 days. Essentially, the development of the bayberry flesh segment involves the structural deformation of the ovary wall. The timing of flesh segment development in bayberry mirrors the findings from fingered citron (*Citrus medica* L. var. *sarcodactylis* Swingle), where finger-like protrusions attached to the primordial ovary form early in carpel development, suggesting that the fruit shape of fingered citron is established before flowering [39]. Therefore, investigations into early fruit morphogenesis should focus on earlier stages of floral organ development.

The determination of endogenous hormones in bayberry flower buds revealed that flesh segment development was closely associated with IAA, JA, ABA, and gibberellins. Significant correlations were observed among these hormones, with the interactions primarily involving JA, IAA, cytokinins, gibberellins, and ABA. The regulatory role of plant hormones in plant development, originating from the spatiotemporal expression of a series of downstream responsive genes, is well-established. For example, research on tomato fruit development has demonstrated that the balance of auxins and gibberellins plays a critical role in determining fruit size and shape, with alterations in the levels of these hormones leading to significant morphological changes [40]. Similarly, studies on peach (*Prunus persica*) have shown that the interaction between auxin and ethylene or ABA influences fruit growth and ripening processes [41,42]. Therefore, in this study, transcriptome data were integrated to identify the key plant hormone signaling pathways in flesh segment development and to predict key genes. Expanding the understanding of these hormonal interactions can provide valuable insights into the mechanisms regulating fruit morphology development in other species.

### 3.2. Prediction of Molecular Mechanisms Regulating Bayberry Flesh Segment Development Through Hormonal Interaction Modulation

The development of columnar fruit tissues in bayberry involves complex hormonal signaling, with auxin and JA playing crucial roles. Transcriptome analysis (GSEA and KEGG) identified the significant upregulation of genes related to these hormonal signaling pathways, including members of the *LAX*, *AUX/IAA*, *JAZ*, and *KANADI* (*KAN*) families, which are crucial for hormone transport and signaling. In this study, WGCNA was used to investigate the mechanisms underlying the hormonal regulation of columnar tissue development and to identify potential regulatory genes. Notably, *CRC* and *SEP* genes, which are the members of the *YABBY* and E-class *MADS-box* gene families, were identified as key regulatory genes. Previous studies have shown that these genes regulate the development of flower organs such as the carpel morphology, and the developmental pattern of the carpel will to some extent affect the final shape of the fruit [43,44]. The development of the flesh segment in bayberry is accompanied by the continued development of floral organs. Our findings suggest they also influence fruit structure, particularly through their interactions within auxin signaling pathways, which are essential for determining fruit shape.

Our analysis revealed a significant reduction in the IAA level during tissue maturation. Could this reduction in auxin content in specific tissue regions lead to an uneven auxin distribution? During fruit shape development, local auxin accumulation can lead to the formation of distinct fruit shapes, such as the elongated fruit neck in cucumber [45] and deformed fruit shape in strawberry [46]. Some fruit shape-regulating genes, such as *SUN*, influence fruit morphology by modulating auxin synthesis, polar auxin transport, and auxin signaling pathways [47]. This highlights the importance of auxin distribution patterns in fruit shape development. The immunofluorescence localization experiments of this study confirmed the specific distribution of auxin within tissues, further supporting our findings. The role of auxin in fruit development is well-established, with high levels of IAA potentially inhibiting fleshy fruit formation [48]. Our results indicated that IAA was highly enriched at the outer edge of columnar tissue. This auxin distribution pattern was crucial for the unique columnar morphology of bayberry fruit. We hypothesized that this pattern is supported by the expression of *LAX* family members involved in auxin signaling. Additionally, *KAN* family members, such as *KAN1* and *KAN4*, are implicated in polar growth [49]. Their detection in our analysis supports the idea that hormonal concentration gradients lead to morphological variations. In the auxin signaling pathway, *KAN1* not only inhibits auxin synthesis and transport but also interacts with ARFs to regulate downstream auxin responses [50]. It is noteworthy that the *KAN1* promoter mutation in the *leaf adaxialized 1* (*lad1*) mutant of rice (*Oryza sativa* L.) significantly increases the JA level [51]. The interaction between *KAN* family members and plant hormones further highlights the intricate balance among plant hormone signaling pathways.

This study indicated that JA played a significant role in fruit development, as evidenced by the upregulation of *JAZ* family members. JAZ proteins act as negative regulators in the JA signaling pathway and interact with downstream targets, including *AP2* family members, which are also upregulated [52,53]. Some JAZ proteins can interact with *AP2* family members to regulate floral organ development in plants [54]. The expression of *JAZ* is influenced not only by JA levels but also by auxin levels. Auxin treatment can stimulate the expression of *JAZ1* in *Arabidopsis thaliana*, where *JAZ1* expression exhibits auxin-induced characteristics [55]. This indicates a complex interaction between JA and auxin in the regulation of tissue development.

This study underscores the critical role of hormonal regulation in the development of columnar fruit tissues in Chinese bayberry (*Myrica rubra*). The interplay between auxin and JA, as well as their interactions with key regulatory genes such as *CRC*, *SEP*, *LAX*, *JAZ*, and *KAN*, provides insights into mechanisms regulating fruit development. Understanding these hormonal pathways enables the optimization of cultivation techniques and the selection of varieties with desirable traits (such as improved fruit shape and size), thus potentially impacting agricultural practices and breeding programs significantly.

Despite its potential contributions, this study has several limitations. The small sample size and the controlled experimental conditions may not fully reflect the complexity of field environments. To enhance breeding precision, in future studies, researchers should focus on identifying specific genetic markers associated with these hormonal pathways. Additionally, it is essential to validate these findings under various environmental conditions and fruit species to provide broader insights into fruit development processes. In addition, investigating the genetic mechanisms underlying hormonal regulation will deepen our understanding of flesh segment development and enhance its practical applications in agriculture.

## 4. Conclusions

Paraffin sectioning experiments clarified the timing and pattern of flesh segment development. Cellular observations revealed distinct stages, including the formation of a primordium, the development of a simple columnar structure, and the formation of the primary flesh segment. Measurements of endogenous hormone levels at these three stages indicated that flesh segment development was closely associated with JA, auxin, ABA, and gibberellins.

**Figure 15:**
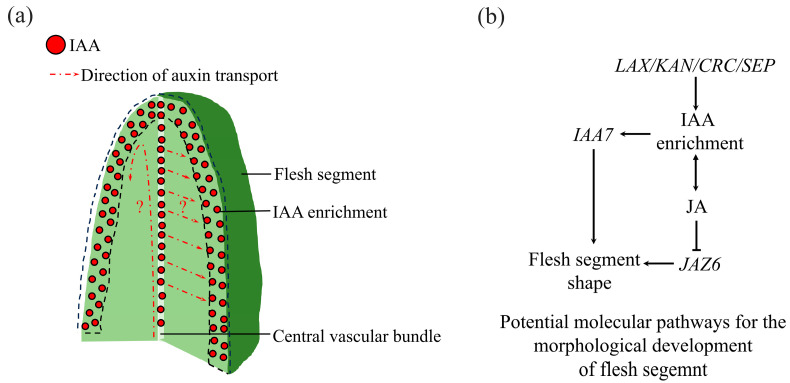
The mechanism of morphological development of flesh segment. (**a**) Distribution pattern of IAA in flesh segment. The red dots represent IAA, the black dashed box indicates the enriched area of IAA, and the red dashed arrow represents the possible transport direction of IAA. (**b**) Potential molecular mechanisms underlying the morphological development of flesh segment. IAA is enriched in the outer cell layer of the flesh segment, which may be the reason for the unique morphology of the flesh segment. In addition, IAA is also enriched in the central vascular system, where there may be transport of IAA. It is speculated that there are two possible transport directions: one is that IAA is transported through the vascular system to the top of the flesh segment and then distributed to the outer edge cell layer, and the other is that IAA is transported horizontally within the vascular system and directly distributed to the outer edge cell layer. Molecular pathways for predicting the development of flesh segment morphology based on transcriptome analysis of genes selected through functional analysis and hormone pathway analysis. According to the results, it was found that there is a strong correlation between auxin and jasmonic acid during the development of the flesh segment. It is speculated that the development of the flesh segment may involve the joint regulation of two hormone pathways, and the regulatory network may involve *LAX*\*CRC*\*SEP* as the upstream, *IAA7* as the downstream auxin regulatory pathway, and *JAZ6* as the responsive jasmonic acid regulatory pathway.

Auxin distribution analysis indicated that auxin was not evenly distributed in the flesh segment; it was primarily concentrated at the apex and outer cells, with additional accumulation in the central vascular bundles. This specific pattern might be related to the elongated morphology of the flesh segment (Figure 15a).

To validate the transcriptome data, we performed a comparative analysis to confirm their accuracy. The results indicated that genes involved in carpel development, such as *CRC* and *SEP*, responded to the JA and auxin signaling pathways during flesh segment development. GSEA and KEGG pathway enrichment analysis revealed that genes related to the JA and auxin signaling pathways played significant roles in flesh segment development.

The *LAX*, *AUX/IAA*, *JAZ*, and *KAN* gene families were crucial in the JA and auxin signaling pathways. The qRT-PCR analysis of the expression levels of genes associated with flesh segment development showed that *CRC*, *SEP1*, *SEP3*, *IAA7*, and *JAZ6* exhibited upregulation trends in both the ‘Biqi’ and ‘Zaojia’ varieties. We speculated that the possible molecular mechanisms underlying the morphological development of flesh segment were shown in Figure 15b.

Future research should focus on the functional validation of these gene family members and a comprehensive investigation into the regulatory mechanisms of the hormone network involved in flesh segment development. Additionally, incorporating metabolomic studies is essential for understanding the metabolic changes associated with flesh segment development. Furthermore, the auxin transport-related genes identified in the transcriptome data warrant further exploration for clarifying their roles in flesh segment development.

## 5. Materials and Methods

### 5.1. Plant Materials and Sampling Methods

In Zhejiang Province, Chinese bayberry is primarily cultivated in regions such as Ningbo, Jinhua, and Taizhou. The ‘Biqi’ variety is the most commonly grown cultivar and represents a typical genetic resource. The ‘Zaojia’ variety, a bud mutation of ‘Biqi’, is characterized by early flowering and early fruiting traits [56]. Selecting ‘Biqi’ and ‘Zaojia’ as research subjects for flesh segment development is representative and allows an investigation into how the early flowering and fruiting characteristics of ‘Zaojia’ impact flesh segment development.

This study utilized perennial bayberry trees of the ‘Biqi’ and ‘Zaojia’ varieties from the Qixingshan Mingguo Orchard in Manjian Town, Zhejiang. These trees were grown in plastic greenhouses. Bayberry trees of the same age were selected, and 3–4 branches with strong and uniform growth were chosen from the outer edges of the tree crown in various directions. The most developed female bayberry flower buds from these branches were collected weekly from September 2021 to July 2023. A portion of the collected flower bud samples was preserved in FAA fixative solution for paraffin sectioning to analyze tissue and cell structure. The remaining samples were stored at −80 °C for transcriptome sequencing and endogenous hormone determination experiments.

### 5.2. Morphological Observation and Cytological Paraffin Sectioning of Bayberry Flower Buds

Fresh female bayberry flower buds were collected at various time intervals and photographed using a super-depth-of-field microscope to observe their morphology. The flower buds were then immersed in FAA fixative solution and processed for paraffin sectioning. The formation of flesh segments in bayberry was observed using an inverted microscope, and the cytological characteristics of the flesh segment development process were recorded. Each stage of flesh segment development was carefully documented with detailed outlines and photographs. Significant events in flesh segment development were correlated with the sampling periods, and the morphological characteristics of bayberry flower buds were compared. This comprehensive approach facilitated the identification of morphological features required for subsequent experiments on the determination of endogenous hormone levels.

### 5.3. Determination of Endogenous Hormone Levels in Bayberry Flower Buds

Following cytological and morphological comparisons, samples of bayberry flower buds at various flesh segment development stages were meticulously dissected to remove the bracts, isolating only the pistil and peduncle parts. These dissected parts were immediately frozen in liquid nitrogen and ground into fine powder. Approximately 50–100 mg of the dry powder was placed in a 1.5 mL centrifuge tube, with three biological replicates prepared for each sample group. The levels of endogenous hormones were then analyzed using high-performance liquid chromatography (HPLC) [57]. The endogenous hormone levels in each sample group were calculated using Microsoft Excel software. SPSS 21 (SPSS Science, Chicago, IL, USA) was used to perform one-way analysis of variance (ANOVA) on the data, significant differences were analyzed using Duncan’s test. The levels of endogenous hormones at different stages of flesh segment development were visualized using bar charts created using SigmaPlot 12.5. Correlations between flesh segment development stages and endogenous hormone levels were analyzed using SPSS 21, using Pearson correlation test.

### 5.4. Transcriptome Sequencing

The flower buds of the ‘Biqi’ and ‘Zaojia’ varieties were collected at different stages of flesh segment development and used as experimental materials. Transcriptome sequencing was conducted to identify the key hormonal signaling pathways and genes involved in flesh segment development. The samples were labeled as follows: BS1, BS2, and BS3 corresponded to ‘Biqi’ flower buds collected at the stages of no flesh segment development, flesh segment primordium formation, and primary flesh segment formation, respectively. Similarly, ZS1, ZS2, and ZS3 corresponded to ‘Zaojia’ flower buds collected at the same respective stages. For each Chinese bayberry variety and flesh segment development stage, 3 biological replicates, with each being approximately 0.5 g, were prepared, totaling 18 samples. RNA was extracted using the Trizol C reagent kit (Invitrogen, Thermo Fisher, Shanghai, China Ageney). RNA concentrations were assessed using a NanoDrop 2000 spectrophotometer (Thermo Fisher, Shanghai, China Ageney), while RNA integrity was evaluated using an Agilent 2100 Bioanalyzer/LabChip GX system (Agilent, Beijing, China Ageney). A sequencing library was prepared using the NEBNext^®^ Ultra™ RNA Library Prep Kit (New England Biolabs, Beijing, China Ageney). Index encoding was added to the attribute sequence of the sample, and sequencing was conducted using the TruSeq PE Cluster Kit v4-cBot-HS platform (Illumina, San Diego, CA, USA). The sequencing was performed by Biomarker Biotechnology (Beijing, China). The reference genome of Chinese bayberry was obtained from the NCBI database (PRJNA93699, https://www.ncbi.nlm.nih.gov/bioproject?term=PRJNA936999&cmd=DetailsSearch, accessed on 21 February 2023), and the sequencing results were deposited in the NCBI database (PRJNA1152707, https://www.ncbi.nlm.nih.gov/bioproject/1152707, accessed on 26 August 2024).

### 5.5. DEG Screening

Genes differentially expressed at different stages of flesh segment development were identified using the DESeq2 package in R software version 4.3.3. (FDR < 0.01, |Fold Change| > 1.0).

### 5.6. WGCNA Analysis

WGCNA was used to construct gene co-expression networks. The gene expression matrix was converted into an FPKM matrix, and analysis was performed using the WGCNA package in R software [58]. The ‘pickSoftThreshold’ function was used to calculate the soft threshold, ensuring a scale-free network distribution. When the scale-free fit index reached 0.85, the parameter at which the fitting curve first approached this index was selected as the soft threshold. A gene co-expression network was then constructed. Modules were divided using the dynamic tree-cutting method and the TOM matrix, and the module eigengene (ME) was calculated. Clustering was performed, and modules with over 70% similarity were merged. Genes with similar expression profiles were clustered into the same module. Genes within the same module may have similar biological functions [59]. Different stages of flesh segment development were assigned different values: 1 for the pre-flower bud stage with no flesh segment development, 2 for the stage of flesh segment primordium formation, and 3 for the stage of primary flesh segment formation. These values were integrated with the corresponding data on endogenous hormone levels (such as auxin and JA levels) at different stages of flesh segment development. ME values were calculated and correlated with traits observed at different stages of flesh segment development. A correlation matrix was constructed to identify key gene modules involved in flesh segment development. Based on the correlations of flesh segment development stages with endogenous hormone levels, key plant hormone signaling pathways responsive to these modules were inferred. Then, the functional annotation of related members was performed using BLASTp through the Chinese bayberry database (http://www.bayberrybase.cn/, accessed on 10 August 2024).

### 5.7. GSEA Analysis

GSEA was conducted to enrich functionally annotated genes within a background gene set, identify important biological pathways associated with phenotypes, and determine the co-expression trends of genes within each annotated gene set. The analysis was performed using GSEA software (https://www.gsea-msigdb.org/gsea/index.jsp, accessed on 20 August 2024) [60]. DEGs associated with flesh segment development were mapped to gene sets using the Gene Ontology (GO) database. The enrichment of gene set members at both ends of the background gene set (comprising all genes from the undeveloped and developed flesh segment groups, unfiltered by FDR or Fold Change) was calculated. Cumulative statistics were used to obtain the enrichment score (ES) for each gene set, which was subsequently normalized (NES). The NES value was used to determine the trend of coordinated gene expression changes within each gene set and their impact on flesh segment development. The transcriptome FPKM values were log-transformed and standardized, and TBtools version 2.125 was used to generate a heatmap of gene expression for key members involved in the flesh segment development process.

### 5.8. KEGG Pathway Enrichment Analysis

KOBAS software version 2.0 was used to perform KEGG pathway enrichment analysis on DEGs, revealing the top 20 enriched KEGG pathways [61]. Generate a heatmap of gene expression within the relevant pathways using TBtools.

### 5.9. Quantification of the Expression Levels of Genes Involved in Flesh Segment Development Using qRT-PCR

Based on comprehensive transcriptomic analyses, combined with expression level trends, family identification, and functional annotation (especially gene families with plant hormone signaling transduction function and reproductive organ morphological development regulation function), key genes involved in flesh segment development were predicted. The expression levels of genes identified in the transcriptome were quantified by qRT-PCR, with *Actin* used as the reference gene. Primers were designed using Primer Premier 5.0 and synthesized by Shangya Biotechnology (Hangzhou, China). The primer sequences are shown in Table S2. The SYBR^®^ Premix Ex TaqTM PCR Kit (Aidlab, Beijing, China) and a QuantStudio 1 fluorescence quantitative PCR instrument (Applied Biosystems, Waltham, MA, USA) were used for qRT-PCR validation. The data were analyzed using the 2^−ΔΔCt^ method, and the results were visualized using Origin software version 2022.

### 5.10. Immunofluorescence Localization Analysis of Auxin Distribution

Fresh young bayberry fruits were sampled and immersed in Optimal Cutting Temperature (OCT) Compound. The samples were placed under vacuum conditions (–0.09 MPa) for 25 min to allow the embedding medium to fully infiltrate the tissue gaps, ensuring the absence of air bubbles that could negatively affect the quality and integrity of the sections. After embedding, the samples were cryosectioned, with the section thickness set to 15–20 µm. The tissue sections were stored at 4 °C in a refrigerator. A 1× PBS buffer (NaCl 0.8 g/100 mL, KCl 0.02 g/100 mL, Na_2_HPO_4_·H_2_O 0.358 g/100 mL, and KH_2_PO_4_ 0.024 g/100 mL) was prepared, filter sterilized, and set aside. A 5% skimmed milk solution in the 1× PBS buffer was prepared for blocking. A micropipette was used to gently add the 1× PBS buffer to wash away the OCT Compound from the tissue surface. The tissues were then incubated with the 5% skimmed milk solution for 1 h. The primary antibody (a monoclonal anti-auxin antibody produced in mice, SIGMA, Shanghai, China) was diluted at a 1:30 ratio in the 5% skimmed milk solution and applied to the tissue surface. The tissues were incubated overnight at 4 °C in the dark. After incubation, the samples were washed twice with the 1× PBS buffer for 10 min each. The secondary antibody (goat anti-mouse IgG FITC, Biosharp, Hefei, China) was diluted at a 1:100 ratio in the 5% skimmed milk solution, applied to the tissue surface, and incubated for 1 h. The samples were washed again twice with the 1× PBS buffer for 10 min each. Finally, the fluorescence was observed under a laser confocal microscope with an excitation wavelength of 488 nm. The above methods are improved based on reference [62].

## Figures and Tables

**Table 1:** GO biological functions of gene sets identified in the GSEA analysis of ‘Biqi’.

ID	Description	NES	*p*-Value	*q*-Value
GO:0009653	anatomical structure morphogenesis	1.655262799	0.0017	0.062
GO:0099402	plant organ development	1.739543974	0.0017	0.062
GO:0006629	lipid metabolic process	1.759558940	0.0017	0.062
GO:0048608	reproductive structure development	1.471478387	0.0017	0.062
GO:0061458	reproductive system development	1.471478387	0.0017	0.062
GO:0034220	ion transmembrane transport	1.482030812	0.0017	0.062
GO:0008610	lipid biosynthetic process	2.129872215	0.0017	0.062
GO:1905392	plant organ morphogenesis	2.159306723	0.0017	0.062
GO:0048827	phyllome development	1.855942933	0.0018	0.062
GO:0044255	cellular lipid metabolic process	1.935717388	0.0018	0.062
GO:0048367	shoot system development	1.639307136	0.0018	0.062
GO:0015849	organic acid transport	1.750069925	0.0018	0.062
GO:0046942	carboxylic acid transport	1.750069925	0.0018	0.062
GO:0003002	regionalization	1.938039429	0.0018	0.062
GO:0090696	post-embryonic plant organ development	2.047266494	0.0018	0.062
GO:0006721	terpenoid metabolic process	2.208318557	0.0019	0.062
GO:0015979	photosynthesis	1.767283950	0.0019	0.062
GO:0048438	floral whorl development	2.043995091	0.0019	0.062
GO:1905393	plant organ formation	2.237432283	0.0019	0.062
GO:0090698	post-embryonic plant morphogenesis	2.277590511	0.0018	0.062

**Table 2:** GO biological functions of gene sets identified in the GSEA analysis of ‘Zaojia’.

ID	Description	NES	*p*-Value	*q*-Value
GO:0009266	response to temperature stimulus	1.493651097	0.0014	0.067
GO:0044550	secondary metabolite biosynthetic process	1.696530376	0.0015	0.067
GO:0009408	response to heat	2.263549549	0.0015	0.067
GO:0009636	response to toxic substance	1.798591204	0.0015	0.067
GO:0015979	photosynthesis	2.247538609	0.0016	0.067
GO:0048467	gynoecium development	2.125138952	0.0016	0.067
GO:0090697	post-embryonic plant organ morphogenesis	1.902950628	0.0016	0.067
GO:0019684	photosynthesis, light reaction	2.26312824	0.0016	0.067
GO:0042542	response to hydrogen peroxide	1.963222347	0.0016	0.067
GO:0048440	carpel development	2.206113474	0.0017	0.067
GO:0048444	floral organ morphogenesis	2.155025208	0.0017	0.067
GO:0035670	plant-type ovary development	1.930760936	0.0017	0.067
GO:0048481	plant ovule development	1.930760936	0.0017	0.067
GO:0009767	photosynthetic electron transport chain	1.94653995	0.0017	0.067
GO:0009765	photosynthesis, light harvesting	2.194935382	0.0017	0.067
GO:0048449	floral organ formation	2.278441944	0.0017	0.067
GO:0046685	response to arsenic-containing substance	2.059134253	0.0018	0.067
GO:0009768	photosynthesis, light harvesting in photosystem I	2.014041361	0.0018	0.067
GO:0010093	specification of floral organ identity	2.062085444	0.0018	0.067
GO:0090701	specification of plant organ identity	2.062085444	0.0018	0.067

## Data Availability

Raw sequence data generated for this study are available at the National Center for Biotechnology Information (NCBI) under BioProject accession number: PRJNA1152707.

## Supplementary Materials

The following supporting information can be downloaded at: https://www.mdpi.com/article/10.3390/plants14040571/s1, Figure S1: Transcriptome validity test results for (a) ‘Biqi’ and (b) ‘Zaojia’; Figure S2: Gene identified in modules associated with flesh segment development. K-means clustering was performed on the interaction network, with the same color representing predicted interactions within the same network; Figure S3: GSEA analysis of DEGs involved during flesh segment development in (a) ‘Biqi’; (b) ‘Zaojia’; Table S1: Quality assessment of sequencing data; Table S2: Primers used for qRT-PCR analysis.
