# Supplementary material for: Comprehensive Analysis of Hormonal Signaling Pathways and Gene Expression in Flesh Segment Development of Chinese Bayberry (Myrica rubra)
Source: Plants (Basel). 2025 Feb 13;14(4):571. doi: 10.3390/plants14040571 (PMC11858897; doi:10.3390/plants14040571)
Supplement: Supplementary file 1 [file plants-14-00571-s001.zip › Table S1.pdf]

Table S1. Quality assessment of sequencing data

Sample	Clean reads	GC Content	%≥Q30	Mapped reads
BS1-1	21711510	46.34%	97.02	41,506,203 (95.59%)
BS1-2	23995216	46.3%	96.52	45,060,479 (93.89%)
BS1-3	23706438	46.43%	96.5	45,395,081 (95.74%)
BS2-1	27988769	46.4%	96.05	53,398,216 (95.39%)
BS2-2	23438022	46.29%	96.52	44,933,202 (95.86%)
BS2-3	21925621	46.35%	98.21	42,441,340 (96.78%)
BS3-1	23281113	46.58%	96.6	44,030,243 (94.56%)
BS3-2	21133022	46.33%	98.1	40,376,001 (95.53%)
BS3-3	23112070	46.31%	98.61	44,510,754 (96.29%)
ZS1-1	23000271	46.64%	97.38	43,390,637 (94.33%)
ZS1-2	22903855	46.82%	97.52	43,784,668 (95.58%)
ZS1-3	19180095	46.59%	98.2	37,419,238 (97.55%)
ZS2-1	20703201	46.38%	98.26	40,327,318 (97.39%)
ZS2-2	22243049	46.5%	97.23	43,201,869 (97.11%)
ZS2-3	22890503	46.57%	98.13	44,650,316 (97.53%)
ZS3-1	19671486	46.07%	97.92	38,152,987 (96.98%)
ZS3-2	19123254	46.09%	98.03	36,995,079 (96.73%)
ZS3-3	22596262	46.5%	97.15	41,990,832 (92.92%)
