# Supplementary material for: Comprehensive Analysis of Hormonal Signaling Pathways and Gene Expression in Flesh Segment Development of Chinese Bayberry (Myrica rubra)
Source: Plants (Basel). 2025 Feb 13;14(4):571. doi: 10.3390/plants14040571 (PMC11858897; doi:10.3390/plants14040571)
Supplement: Supplementary file 1 [file plants-14-00571-s001.zip › Table S2.pdf]

Table S2. Primers used for qRT-PCR analysis

Gene	ID	Forward primer (5'-3')	Reverse primer (5'-3')	Tm(℃)	Fragment length/bp
IAA1	MrChr3G26780	ATGTCGCCGGAAAATGG	CGAAAATCCACGTTTCATGC	55.5 ℃	150 bp
IAA7	MrChr3G32340	ATGGAAGTTAGCCGGAAAATTA	AGTCTCAGAGAACCCTCTCTTTCC	55.0 ℃	150 bp
IAA9	MrChr8G25860	TCCGTTGACAGCTTATGTCGTA	CAGACTTCCCGATCTCTTTCAG	55.0 ℃	200 bp
IAA16	MrChr8G10460	ATGGGTACCAAGTTGGAGGTAG	AACGGTTTCCGAAAACCCT	56.0 ℃	150 bp
GH3.1	MrChr5G29170	ATCCAGCGTATTGCTAATGGTGATC	TTCCCCTTGTCCAGATCCTGGA	57.0 ℃	200 bp
GH3.2	MrChr7G01850	ACGAGGATCTTCAGCCCGCAAT	CACATAAAGGTTCATTACTGGCATG	57.0 ℃	200 bp
GH3.6A	MrChr4G05370	AGATGAAGTTCAGCAGGGTGTTCTG	GATTCGAGCAGAGGATTGGGG	58.0 ℃	200 bp
GH3.6B	MrChr6G20730	AATGTTGTTCTTAGTATTGATTCGG	GAGGGAGGAACTAGGGTTTCG	58.0 ℃	200 bp
JAZ2	MrChr3G29740	ACCTGAGAAGTCCAATTTTGCA	GTTTCCATGTTTGATAATAAATCCA	56.5 ℃	150 bp
JAZ6	MrChr7G25600	AATACATCTCATCCAAAAGCTCAAG	CTGACTGGGCGCCAGAATGCGAAGA	55.0 ℃	150 bp
JAZ8	MrChr2G18530	ATGAGGAGGAATTGCAACTTG	TTGCTGTTCCTCTTGGTTCG	56.0 ℃	150 bp
JAZ10A	MrChr7G09130	AAGATCAACCCTGAGGTTTTG	ACCGGGCCTGAAGGAAGGAGTATAG	56.0 ℃	150 bp
CRC	MrChr7G19380	AGAAAGTCTCTATGGACCTGGTTCC	CAGGGGAAGATCATGACATTGC	59.5 ℃	200 bp
SEP1	MrChr6G06270	TGGATGAGATTAGCGGAAGAAAT	GAACCCACTGACTTGTTGGG	59.0 ℃	200 bp
SEP3	MrChr7G08740	GATATCACGTTAATTCACTTCAACT	TGGCAACCACCCTGGCAT	57.5 ℃	200 bp
LAX2	MrChr2G03550	ATAGCTTGTGCGAGTAACATCTACT	TTCTGCCCATGAACGAAGG	58.5 ℃	200 bp
LAX3	MrChr7G29370	GATTTCAGGAATCATGTCATTCAGT	CAGGCGCCAAAGATGTATGT	58.0 ℃	200 bp
KAN1	MrChr6G07090	GAAGGGATTTTCATTGAACCTT	AGCTCAGTATGAGCTTGGGAAT	58.5 ℃	200 bp
KAN4	MrChr3G08110	CTAGGCCAGAACCCTATAATGG	AAGAAGCTGTTTTCATGGCTTA	56.0 ℃	200 bp
Actin	MrChr4G09970	GGGACAGAAAGATGCCG	TCCATGTCATCCCAGTTGCTAA	55.0 ℃	108 bp
